# Interventional tracheoscopy for management of intratracheal plasmacytoma in a dog

**DOI:** 10.1007/s11259-026-11478-9

**Published:** 2026-08-28

**Authors:** João Igor Oliveira Malheiro, Kesia Bandeira da Silva, Iroleide Santana de Jesus, Adriana Vasconcelos Nobre de Araújo, Pedro Soares Bezerra Júnior, Sandla Freitas da Silva, Francisco Décio de Oliveira Monteiro, Roberta Martins Crivelaro, Roberto Thiesen, Pedro Paulo Maia Teixeira

**Affiliations:** 1 Veterinary Medicine Institute, Federal University of Pará, Castanhal, Pará Brazil; 2 Federal Institute of Tocantins (IFTO), Campus Araguatins, Araguatins, Tocantins Brazil

**Keywords:** Dog, Extramedullary plasmacytoma, Interventional endoscopy, Intraoperative haemorrhage, Tracheal tumour

## Abstract

Intraoperative haemorrhage during interventional tracheoscopy for intratracheal masses can pose a life-threatening challenge. This report emphasizes the technical management of severe bleeding during endoscopic excision of a rare tracheal plasmacytoma in a dog, highlighting the importance of immediate replacement of the endoscope with a larger-calibre device and use of bipolar electrosurgical forceps placed parallel to the endoscope. An 8-year-old male Standard Poodle presented with a two-month history of nonproductive cough and exertional dyspnea. Cervical radiography revealed a nodular mass (1.21 × 1.13 cm) attached to the dorsal wall of the cervical trachea. During anesthetic induction for diagnostic and therapeutic tracheoscopy, the endotracheal tube inadvertently compressed the mass, causing substantial haemorrhage with imminent risk of blood aspiration and airway obstruction. The endoscope was immediately replaced with a larger-calibre (5.9 mm) device to improve lavage and suction. After removing the endotracheal tube to permit parallel instrument placement, initial attempted resection using a polypectomy snare failed due to recurrent bleeding. Bipolar electrosurgical forceps were then introduced parallel to the endoscope, directly grasping and coagulating the tumour base, which achieved hemostasis and enabled gross endoscopic excision (macroscopic removal of the visible tumour). The dog recovered without respiratory distress and was discharged the same day. Histopathology and immunohistochemistry confirmed extramedullary plasmacytoma. Gross endoscopic excision was achieved; histological margins were not assessable due to cautery artefact. The dog remained free of respiratory signs and tumour recurrence at 12-month follow-up.

## Background

Primary intratracheal neoplasms are uncommon in small animals and humans; however, they are associated with high morbidity and mortality, especially when diagnosis is delayed (Nilssen et al. 2023; Huang et al. 2024). In a comprehensive review of tracheal neoplasia in dogs and cats from 1961 to 2024, Berrocal et al. (2025) identified that these tumours account for a small fraction of respiratory tract malignancies. In dogs and cats, squamous cell carcinoma, osteochondroma, and adenoma are the most frequently reported neoplasms at this anatomic site (Behrens et al. 2022; An et al. 2024). In dogs specifically, reported tracheal tumours include osteochondroma, malignant epithelial neoplasms, and plasma cell tumours, with the latter group being among the least frequently documented (Berrocal et al. 2025).

Plasma cell tumours include multiple myeloma and solitary plasmacytoma, and the latter is subdivided into osseous and extramedullary forms; these tumours originate from differentiated B lymphocytes that produce immunoglobulins (Hayes et al. 2007; Mikiewicz et al. 2016). Extramedullary plasmacytoma (EMP) occurs predominantly on the skin of dogs, cats, and horses and accounts for approximately 1.5% of canine cutaneous tumours (Boes and Durham 2017). Although EMP may affect other organs, including the oral cavity, gastrointestinal tract, liver, spleen, lungs, and central nervous system, tracheal involvement is extremely rare. Prior to the retrospective series by Mulka et al. (2025), which reported ten cases of canine laryngotracheal plasma cell tumours (five tracheal and five laryngeal), fewer than ten individual case reports of tracheal EMP had been published (Elmenhorst et al. 2018; Ferronato et al. 2023). The true incidence of tracheal EMP in dogs remains unknown; therefore, epidemiological claims must be interpreted cautiously (Berrocal et al. 2025).

Clinically, dogs with intratracheal neoplasms can have signs consistent with upper respiratory tract obstruction, including nonproductive cough, stridor, dyspnea, exercise intolerance, and episodes of cyanosis, regardless of age, sex, or breed (Ferronato et al. 2023; Galeno et al. 2025). Diagnosis and staging involve clinical and laboratory evaluation combined with diagnostic imaging, such as radiography, ultrasonography, and especially tracheoscopy, allowing direct visualisation of the lesion, sample collection, and therapeutic planning (Joffe et al. 2020; Valente et al. 2024). In a prospective comparison of diagnostic techniques for canine tracheal masses, De Lorenzi et al. (2025a, b) demonstrated that endobronchial needle aspiration, bronchial brushing, and forceps biopsy each have complementary roles in achieving a definitive diagnosis.

Treatment is generally based on surgical resection, with or without chemotherapy, depending on the extent and biological behaviour of the neoplasm. However, tracheal surgery is technically difficult due to restricted anatomic access, the risk of haemorrhage, intraoperative ventilatory instability, and limitations related to dog size and available equipment (Miller et al. 2020). Although open surgical approaches such as tracheal resection and anastomosis have been reported for various tracheal tumours (Behrens et al. 2022), advances in interventional endoscopy have increased therapeutic options by allowing more precise and less invasive procedures, with a potential reduction in complications (Hur et al. 2025; Joffe et al. 2020; Britany et al. 2015). Endoscopic techniques for tracheal masses include polypectomy snares, biopsy forceps, and electrosurgical devices, each with specific advantages and limitations regarding haemorrhage control and margin assessment.

This report describes the interventional endoscopic management of an intratracheal plasmacytoma in a dog. Although the tracheal location of this neoplasm is rare, the primary educational value of this report lies not in the lesion's rarity, but in the practical lessons learned from managing a severe intraoperative haemorrhage during interventional tracheoscopy. The technical strategies employed, immediate endoscope exchange, removal of the endotracheal tube, and bipolar electrosurgical forceps placement, offer a practical rescue approach for similar airway emergencies.

## Case presentation

An 8-year-old intact male Standard Poodle, weighing 12.5 kg, was presented with a two-month history of nonproductive cough and intermittent episodes of dyspnea triggered by physical activity.

On presentation, the dog was quiet but alert. The respiratory rate was 32 breaths per minute (reference range 10–30), and oxygen saturation (SpO₂) measured by pulse oximetry was 94% on room air, indicating mild hypoxemia. Thoracic auscultation revealed increased inspiratory and expiratory effort with referred upper airway noise. Mucous membranes were pink and moist. The remainder of the physical examination was unremarkable. The clinical presentation was consistent with partial upper respiratory tract obstruction.

Hematologic and biochemical parameters were within normal limits. Abdominal ultrasonography revealed no pathological alterations or evidence of metastatic disease. Thoracic computed tomography (CT) was considered but was not performed due to financial constraints and the immediate availability of radiography and tracheoscopy for diagnostic and therapeutic intervention.

The animal was treated as part of a routine clinical procedure at the Veterinary Hospital of the Institute of Veterinary Medicine of the Universidade Federal do Pará (UFPA), Brazil. All diagnostic and therapeutic interventions were performed in accordance with institutional ethical guidelines for animal care. Written informed consent was obtained from the dog's owner prior to the procedure, authorising the diagnostic investigation, the therapeutic tracheoscopy, and the subsequent publication of clinical data and intraoperative images for scientific purposes.

### Diagnostic procedures

**Fig. 1:**
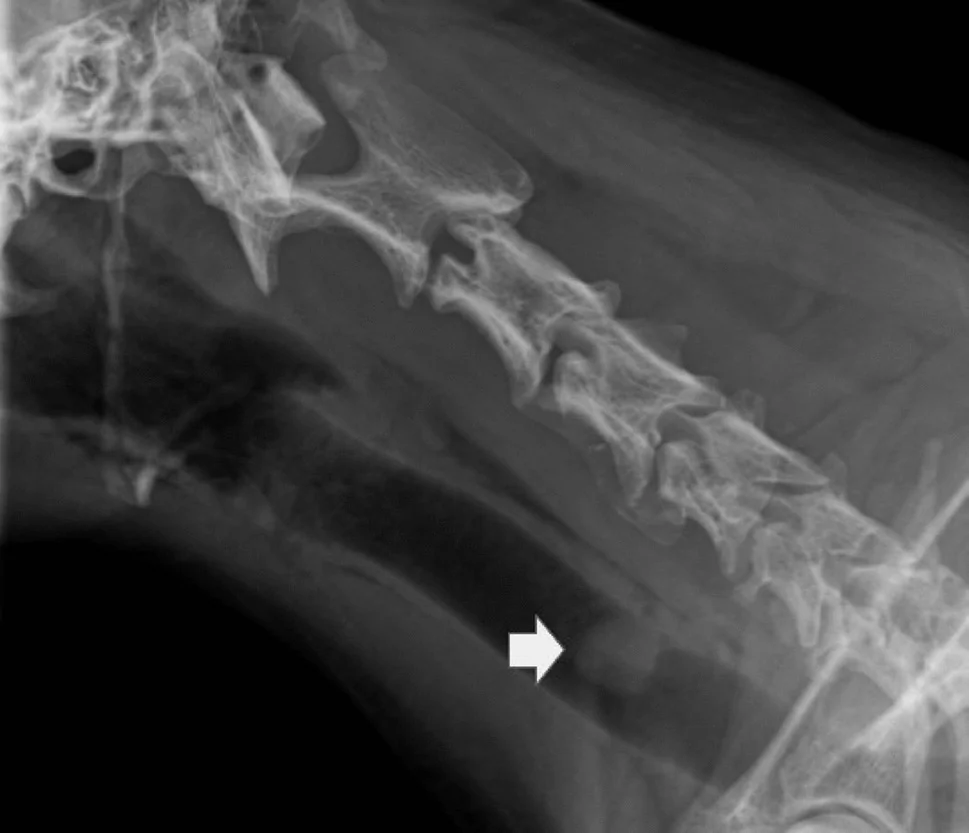
Cervical radiograph in the lateral projection showing a nodular structure attached to the dorsal wall of the trachea in a dog with suspected intratracheal neoplasia (white arrow)

Cervical radiography in the lateral projection identified a moderately radiopaque nodular structure measuring approximately 1.21 × 1.13 cm, attached to the dorsal wall of the cervical trachea (Fig. 1). No other radiographic abnormalities were observed in the cervical structures or the pulmonary parenchyma. Based on this finding, diagnostic and therapeutic tracheoscopy under general anaesthesia was elected as the next step. The dog was classified as ASA II according to the criteria of the American Society of Anesthesiologists (Mayhew et al. 2019).

### Treatment

Premedication consisted of methadone (0.2 mg kg⁻^1^) administered intravenously 10 min before induction. Anaesthesia was induced with propofol (6.0 mg kg⁻^1^ IV to effect) and maintained with total intravenous anaesthesia using propofol (0.4 mg kg⁻^1^ min⁻^1^) and remifentanil (7 μg kg⁻^1^ h⁻^1^) as continuous rate infusions. The animal was initially intubated with a 7.5‑mm endotracheal tube, and diagnostic and therapeutic tracheoscopy was performed.

Complication: During intubation, the tube inadvertently contacted the tracheal mass. A 3.1-mm flexible video endoscope (Kiron Vet®; 100 cm working length; 30° field of view) was initially introduced through the endotracheal tube, revealing that the mass had been compressed and partially collapsed against the dorsal tracheal wall. When the endotracheal tube was moved cranially to clear the surgical field, substantial haemorrhage from the mass occurred, posing an imminent risk of blood aspiration and airway obstruction.

Rescue strategy: The surgical plan was immediately modified. The smaller-calibre endoscope was replaced with a larger (5.9 mm, Kiron Vet®; 40 cm working length; 0° forward view) device to improve lavage and suction capacity. Because the larger endoscope could not pass through the endotracheal tube, the tube was removed and oxygen was supplemented via a nasal catheter. Intermittent blood aspiration was performed with difficulty, achieving temporary hemostasis, preventing pulmonary aspiration, and partially restoring visualization of the base of the lesion. Initial resection with a polypectomy snare was attempted but was ineffective, as bleeding recurred immediately.

**Fig. 2:**
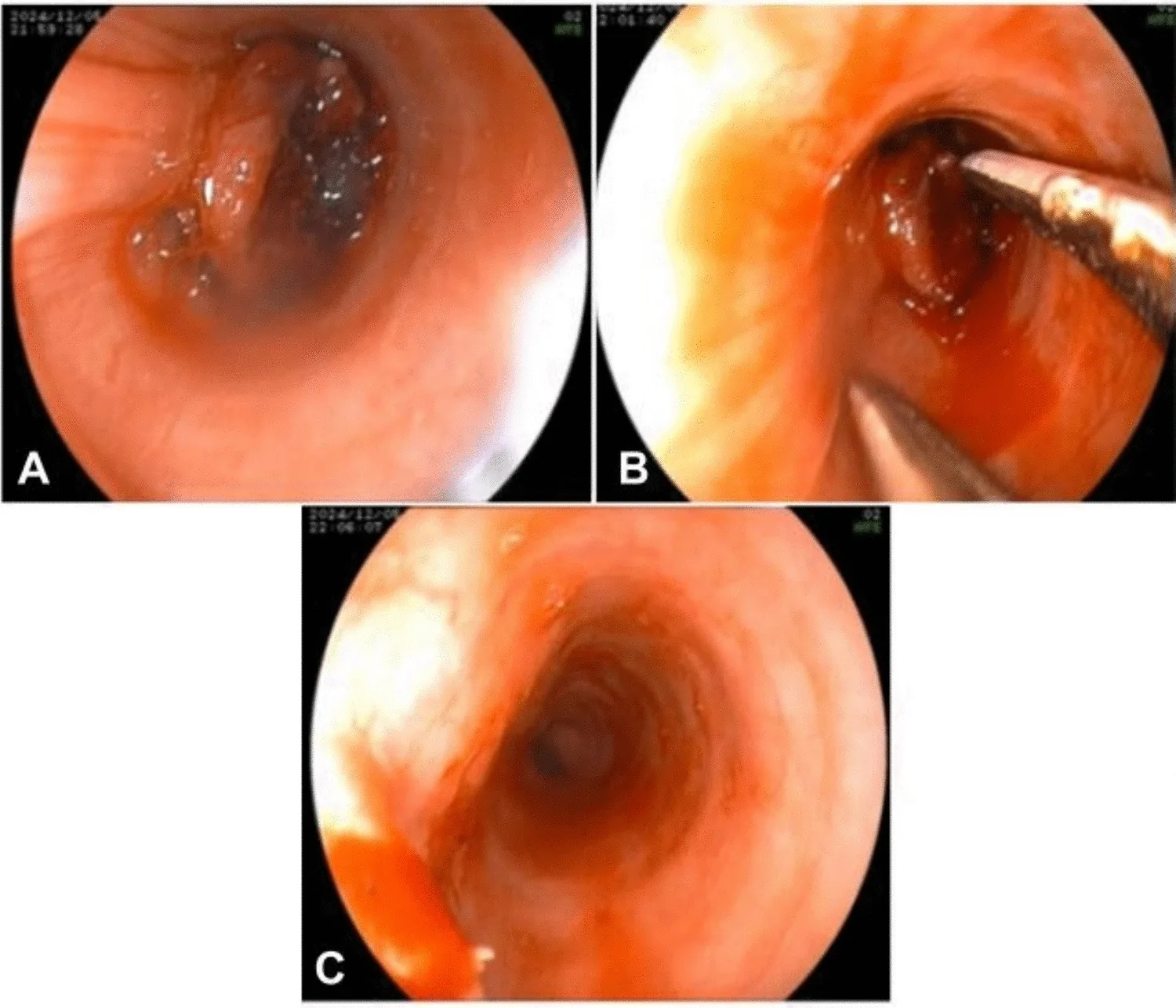
Tracheal mass observed during tracheoscopy in a dog with suspected intratracheal neoplasia. (**A**) Ulcerated nodule attached to the dorsal wall of the trachea. (**B**) Dissection and excision of the lesion using bipolar electrosurgical forceps. (**C**) Final appearance of the tracheal lumen after tumour excision

Definitive excision: Bipolar electrosurgical forceps were then introduced parallel to the endoscope, directly grasping and coagulating the base of the mass. This provided definitive control of haemorrhage and allowed gross endoscopic excision (macroscopic removal of the visible tumour) of the lesion. After removal, the tracheal lumen showed no active bleeding and preserved adjacent mucosa (Fig. 2A–C).

Before removal of the endotracheal tube, controlled mechanical ventilation was used (pressure-limited ventilator, peak inspiratory pressure 12 cmH₂O, rate 10 breaths per minute). After the endotracheal tube was removed to permit parallel placement of instruments, the dog was maintained on spontaneous ventilation with oxygen supplementation (100% oxygen at 2 L/min) delivered via a nasal catheter placed in the ventral meatus of the right nostril. During this phase, SpO₂ was monitored continuously via pulse oximetry (probe placed on the tongue) and remained above 90%; respiratory rate and effort were assessed clinically; end-tidal CO₂ was measured via a side-stream adapter connected to the nasal catheter. No alternative airway device (e.g., laryngeal mask airway, rigid bronchoscope with ventilation port) was used due to the emergency nature of the haemorrhage and the adequacy of the nasal catheter technique for oxygenation. Controlled ventilation was not continued after tube removal, as spontaneous breathing was sufficient to maintain oxygenation while the endoscopist focused on haemostasis. Alternative airway management strategies, including temporary tracheostomy, rigid ventilating bronchoscopy, and jet ventilation, were considered but not implemented.

### Outcomes

The dog recovered satisfactorily from anesthesia and was discharged the same day. Before discharge, the dog was monitored for approximately 6 h in the hospital, during which respiratory rate and effort were assessed every 2 h, with SpO₂ measured continuously via pulse oximetry, remaining above 96% on room air. The owner was instructed to monitor at home for any signs of respiratory distress, cough, or voice change, and to return for reevaluation at 15 days. No postoperative endoscopy or imaging was performed because the dog remained clinically eupneic without cough or dyspnea. Medical management included meloxicam (0.1 mg kg⁻^1^ SID), dipyrone (25 mg kg⁻^1^ BID), and tramadol (4 mg kg⁻^1^ BID). The animal was discharged on the same day.

**Fig. 3:**
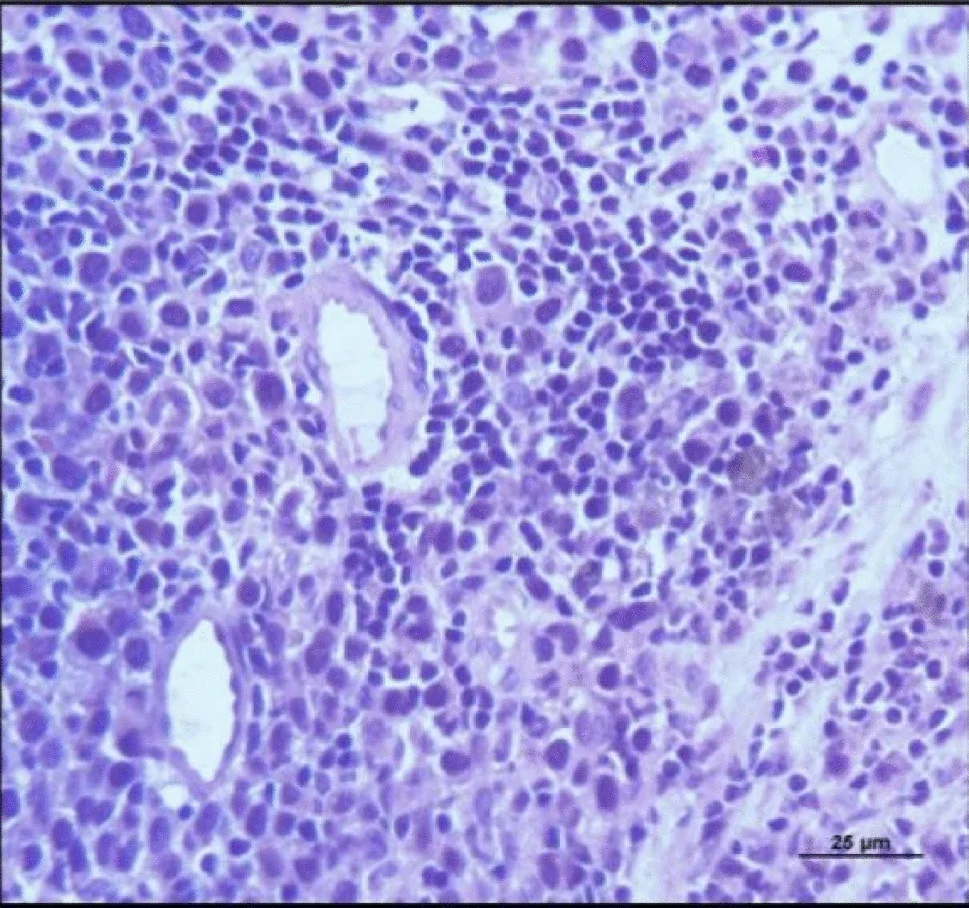
Histopathological photomicrographs of the excised tracheal mass of a dog. Plasmacytoma in the tracheal lumen. Round cells arranged in solid patterns with eosinophilic cytoplasm. Hematoxylin and eosin staining (H&E), 100 × objective

Histopathological and immunohistochemical examination confirmed extramedullary plasmacytoma with a Ki‑67 proliferation index of approximately 45% (Fig. 3). Based on the available diagnostic investigations (three-view thoracic radiography, abdominal ultrasonography, complete blood count, serum biochemistry, and urinalysis), no evidence of metastatic disease or systemic involvement was identified. The dog therefore had findings compatible with a solitary extranodal lesion, which would be consistent with stage I (Holler et al. 2022; Fend et al. 2023). However, the authors acknowledge that formal stage classification per comprehensive plasma cell tumour guidelines would require additional testing (serum protein electrophoresis, bone marrow evaluation, and regional lymph node cytology).

Preoperative coagulation testing (platelet count, prothrombin time, and activated partial thromboplastin time) was not performed. The dog had no history of bleeding tendency, and physical examination revealed no petechiae, ecchymoses, or other signs of coagulopathy. The authors acknowledge this as a limitation.

### Functional outcome

At the 15‑day reevaluation, the dog was in good general condition with no signs of respiratory distress or tumour recurrence. Following gross endoscopic excision, tracheal patency was restored and the presenting clinical signs resolved. The dog was referred for ongoing oncologic follow-up and, to date, remains clinically stable. In this single case, tracheoscopy-guided bipolar electrosurgical excision was feasible and resulted in short-term clinical resolution.

## Discussion

Tracheal extramedullary plasmacytoma is an uncommon neoplasm in dogs, with fewer than ten related cases in a retrospective series (Mulka et al. 2025; Berrocal et al. 2025). This case emphasises haemorrhage precipitated by endotracheal tube contact with a friable intratracheal mass. Prevention of tumour contact during intubation is critical; alternatives include smaller calibre tube intubation under endoscopic guidance or tracheoscopy before intubation (De Lorenzi et al. 2025a, b). CT was not performed, although it could have provided information on tumour dimensions and regional lymph nodes (Berrocal et al. 2025). Haemorrhage control was achieved using a larger-calibre endoscope for suction, removal of the tube, and bipolar electrosurgical forceps for direct coagulation. As noted in the Case Presentation, coagulation testing was not performed; future cases should include platelet count, PT, and aPTT.

The choice between interventional endoscopic techniques and open surgical approaches for tracheal masses depends on tumour characteristics, available equipment, and clinician expertise. Polypectomy snares have been used for removal of tracheal masses but carry a risk of recurrent bleeding (Queen et al. 2010; Howard et al. 2017). Diode laser surgery is an alternative but carries risks of thermal injury (De Lorenzi and Solano-Gallego 2009). Bipolar electrosurgical forceps, as used in this case, offer simultaneous grasping, coagulation, and transection, which may reduce rebleeding compared to snare polypectomy. Tracheal resection and anastomosis remain the gold standard and offer the possibility of complete histological margin assessment and potential cure (Behrens et al. 2022; Miller et al. 2020). However, resection and anastomosis are associated with greater morbidity, longer hospitalisation, risks of anastomotic dehiscence or stricture, and requires specialised surgical expertise. Endoscopic excision offers shorter procedure time, reduced postoperative pain, same-day discharge, and lower cost, but is limited by the inability to assess histological margins and the risk of incomplete resection. Indications for endoscopic excision include small, sessile, intraluminal masses without mural invasion. Open surgery is preferred when complete histological clearance is essential or when the mass is large or invasive (Behrens et al. 2022).

Staging for extramedullary plasmacytoma included three-view thoracic radiography, abdominal ultrasonography, complete blood count, serum biochemistry, and urinalysis. While the available findings are compatible with localised disease without evidence of systemic involvement, the absence of confirmatory testing for systemic plasma cell neoplasia, specifically serum protein electrophoresis, bone marrow evaluation, and regional lymph node cytology, represents a limitation (Mulka et al. 2025). The Ki-67 index of approximately 45% exceeds the typical range for canine extramedullary plasmacytoma (5–25%) (Mulka et al. 2025). However, because complete oncologic staging was not performed and histological margins were not assessable due to cautery artefact, the prognostic implications of this finding remain uncertain. The 12-month recurrence-free interval is encouraging but should be interpreted as an encouraging observation rather than evidence that complete local tumour control was achieved. Late recurrence cannot be excluded, and any suggestion that endoscopic excision mitigated the prognostic impact of the Ki-67 index remains speculative.

The principal contribution of this report lies not in demonstrating the long-term efficacy of endoscopic tumour excision, but in the detailed description of intraoperative decision-making and haemorrhage management during a life-threatening airway complication. The favourable clinical outcome in this dog demonstrates that gross endoscopic excision of an intratracheal plasmacytoma was feasible. However, the technical sequence described, immediate endoscope exchange, removal of the endotracheal tube, and bipolar electrosurgical forceps placement, offers a practical rescue strategy for similar airway emergencies, which represents the primary educational value of this case.

## Conclusions

This case illustrates that interventional tracheoscopy can be a viable option for hemostasis and endoscopic excision of intratracheal neoplasms. Rapid exchange to a larger-caliber endoscope for suction, removal of the tube to permit parallel instrument placement, and bipolar electrosurgical forceps for direct coagulation achieved hemostasis and gross endoscopic excision (macroscopic removal of the visible lesion) of the intratracheal plasmacytoma. A single case does not prove safety or efficacy, yet the technical sequence described here offers a practical rescue strategy for similar airway emergencies.

## Data Availability

No datasets were generated or analysed during the current study.
